# Computational Models in Electroencephalography

**DOI:** 10.1007/s10548-021-00828-2

**Published:** 2021-03-29

**Authors:** Katharina Glomb, Joana Cabral, Anna Cattani, Alberto Mazzoni, Ashish Raj, Benedetta Franceschiello

**Affiliations:** 1 Connectomics Lab, Department of Radiology, Lausanne University Hospital and University of Lausanne (CHUV-UNIL), Lausanne, Switzerland; 2 Life and Health Sciences Research Institute (ICVS), University of Minho, Braga, Portugal; 3 Department of Psychiatry, University of Wisconsin-Madison, Madison, USA; 4 Department of Biomedical and Clinical Sciences ’Luigi Sacco’, University of Milan, Milan, Italy; 5 The BioRobotics Institute, Scuola Superiore Sant’Anna, Pisa, Italy; 6 School of Medicine, UCSF, San Francisco, USA; 7 Department of Ophthalmology, Hopital Ophthalmic Jules Gonin, FAA, Lausanne, Switzerland; 8 CIBM Centre for Biomedical Imaging, EEG Section CHUV-UNIL, Lausanne, Switzerland; 9 Laboratory for Investigative Neurophysiology, Department of Radiology, Lausanne University Hospital and University of Lausanne (CHUV-UNIL), Lausanne, Switzerland

**Keywords:** Electroencephalography, Computational modeling, Multiscale modeling, Clinical applications

## Abstract

Computational models lie at the intersection of basic neuroscience and healthcare applications because they allow researchers to test hypotheses in silico and predict the outcome of experiments and interactions that are very hard to test in reality. Yet, what is meant by “computational model” is understood in many different ways by researchers in different fields of neuroscience and psychology, hindering communication and collaboration. In this review, we point out the state of the art of computational modeling in Electroencephalography (EEG) and outline how these models can be used to integrate findings from electrophysiology, network-level models, and behavior. On the one hand, computational models serve to investigate the mechanisms that generate brain activity, for example measured with EEG, such as the transient emergence of oscillations at different frequency bands and/or with different spatial topographies. On the other hand, computational models serve to design experiments and test hypotheses in silico. The final purpose of computational models of EEG is to obtain a comprehensive understanding of the mechanisms that underlie the EEG signal. This is crucial for an accurate interpretation of EEG measurements that may ultimately serve in the development of novel clinical applications.

## Introduction

Electroencephalography (EEG) has applications in many fields, spanning from basic neuroscientific research to clinical domains. However, despite the technological advances in recording precision, the full potential of EEG is currently not being exploited. One possible way to do so is to use computational models in order to integrate findings from electrophysiology, network-level models (the level of neuroimaging), and behavior (Franceschiello et al. 2018, 2019). The following review has been conceived with the specific goal of targeting a non-expert audience. Indeed this does not constitute an exhaustive review, but we believe that structuring the contents along spatial scales might facilitate the understanding of this broad topic. Furthermore, this review does not cover Brain Computer Interfaces (BCIs), first as they constitute an independent topic itself at the interface between engineering and neuroscience (Bulárka and Gontean 2016). Secondly, the vast majority of BCI works relies on a statistical model of the neural signal, often combined with machine learning approaches, and thus lie outside the scope of this review. Here, our aim is to discuss computational models that integrate cellular behavior at different spatial scales and make explicit links to EEG empirical data. We refer readers interested in BCI to Wolpaw et al. (2000), Schiff (2012), Fouad et al. (2015), Bulárka and Gontean (2016), and for BCI-based EEG paradigms to Abiri et al. (2019).

A model is defined in terms of a set of equations which describe the relationships between variables. Importantly, models exist for different spatial scales (Varela et al. 2001; Deco et al. 2008; Nunez et al. 2019), spanning from the single cell spike train up to macroscopic oscillations. The equations are used to simulate how each variable changes over time, or, in rare cases, to find analytical solutions for the relationships among the variables. The dynamics of the resulting time series are also influenced by a set of parameters, which can either be estimated from available data—for example, a model which simulates the firing of a certain neuron type could contain a time constant estimated from recordings on that type of neuron in rodents—or its value can be varied systematically in an exploratory manner. The goal is to produce time series of variables that can be compared to real data. In particular, one can simulate perturbations to brain activity, be it sensory stimulation, a therapeutic intervention like deep brain stimulation (DBS) or a drug, or a structural change due to the onset of a pathology, like neurodegeneration or a lesion, and predict what would be the resulting alterations observed in neural and clinical data.

An important application of EEG models is in the clinical domain. Psychiatric and neurological disorders impact a growing portion of the population, both as patients and caregivers, and with an enormous cost—both economical and humanitarian—to healthcare systems worldwide (Steel et al. 2014; Vigo et al. 2016; Feigin et al. 2019). One of the main obstacles in advancing patient care is the lack of individual diagnosis, prognosis, and treatment planning (Wium-Andersen et al. 2017). Computational models can be adapted to the individual by setting their parameters according to available data (i.e. either setting the parameter directly, if it is measurable, or looking for the parameter value which results in time series whose dynamics match recorded data). The adjusted parameter(s) can then be related to clinical markers, symptoms, and behavior, allowing for example to discriminate between pathologies. Using models in this personalized manner could provide additional diagnostic features in the form of model parameters and model output, eventually assisting clinicians in diagnosis and treatment planning.

Another obstacle is a general lack of scientific knowledge of disease mechanisms, including the mechanisms by which therapies exert their effect. As an example, DBS is a highly effective treatment for advanced Parkinsonism, in which electric pulses are delivered directly to certain deep brain structures via permanently implanted electrodes. Yet, it is largely unknown how exactly the applied stimulation manages to suppress motor symptoms such as tremor (Chiken and Nambu 2016). This is also because the way in which motor symptoms result from the degradation of dopaminergic neurons in the substantia nigra is not fully understood (McGregor and Nelson 2019). Besides animal models—which have their own ethical issues—in silico models are an indispensable tool for understanding brain disorders. Combining data available from a patient or group of patients with knowledge and hypotheses about mechanisms, a model can be generated which can help test these hypotheses.

Last but not least, models are much cheaper than animal testing or clinical trials. While models will not replace these approaches—at least not in the foreseeable future—they could help to formulate more specific hypotheses and thus, lead to smaller-scale experiments.

Collecting invasive data is not generally possible in humans. EEG (Nunez et al. 2006; Schomer and Lopes Da Silva 2012; Biasiucci et al. 2019) is an extremely versatile technology which allows non-invasive recording of neural activity in behaving humans. EEG is a cheap and portable technology, particularly compared to (f)MRI and MEG. Apart from these cost-efficiency considerations, EEG, like MEG, is a direct measure of the electromagnetic fields generated by the brain, and allows millisecond-precision recordings, thus giving access to rich aspects of brain function which can inform models in a way that e.g. fMRI cannot (see “Electroencephalography” section for more details). In general, using different complementary sources of data to construct and validate a model will lead to better model predictions, as each recording technique has its own strengths and weaknesses, and a multimodal approach can balance them.

In our opinion, there are mostly two reasons why EEG has not been used more extensively in modeling studies, and particularly in a clinical context. First, there are numerous technical problems which make the processing and interpretation of EEG data challenging. EEG—like MEG—is measured on the scalp, and the problem of projecting this 2D-space into the 3D-brain space arises (Michel and Brunet 2019). While multiple solutions exist for this inverse problem, it is unclear which one is the best and under which circumstances (Hassan et al. 2014; Mahjoory et al. 2017; Hedrich et al. 2017). It is important to point out that the goal of source reconstruction is not necessarily to mimic the underlying brain activity, but rather to identify the spatial origins of the signal recorded at the scalp. The question of what constitutes a “source” is still controversial in this context, and the definition depends on the spatial scale (Nunez et al. 2019). Thus, source activity has to be interpreted carefully, taking into account varying degrees of abstraction. Since this is a complex problem involving biophysical mechanisms, we leave this topic aside and recommend the review by Nunez et al. (2019), which addresses the problem of source localization by means of computational models of neural activity on different spatial scales.

EEG data require extensive preprocessing, e.g. removal of artifacts due to movements, eye blinks, etc., but these steps are far from being standardized, and many options exist. The recently started EEG-BIDS effort (Pernet et al. 2019) is a step towards the direction of standardization of EEG data and should facilitate, alongside with the much larger amount of publicly available data, studies that systematically evaluate the impact of preprocessing steps and compare source reconstruction algorithms. As the interest in EEG rises, the need to resolve these issues will trigger larger efforts that will benefit the entire community.

The second obstacle to a more routine usage of computational models in EEG research, which we hope to address in this review, is that such models usually require an understanding of the mathematics involved, if only to be able to choose the model that is useful for the desired application. Both variables and parameters are not always clearly related to quantities which can be measured in a clinical or experimental context, and more generally, models need to be set up in such a way that they meet existing clinical demands or research questions.

The contribution of this paper is threefold. First, this article summarizes computational approaches at different spatial scales in EEG, targeting non-experts readers. To the best of our knowledge, this paper represents the first review on this topic. Second, we will point out several ways in which computational models integrate EEG recordings, by using biologically relevant variables. Third, we discuss the clinical applications of computational models in EEG which have been developed. The field is greatly expanding and contains promising advancements both from research and clinical standpoints. We believe that this overview will make the field accessible for a broad audience, and indicate the next steps required to push modeling of EEG forward.

## Electroencephalography

EEG is a non-invasive neuroimaging technique that measures the electrical activity of the brain (Nunez et al. 2006; Schomer and Lopes Da Silva 2012; Biasiucci et al. 2019). EEG recordings have been a driver of research and clinical applications in neuroscience and neurology for nearly a century. EEG relies on the placement of electrodes on the person’s scalp, measuring the postsynaptic potentials of pyramidal neurons (Tivadar and Murray 2019; Lopes da Silva 2013). EEG does not directly measure the action potentials of neurons, though there are some indications of high-frequency oscillations being linked to spiking activity (Telenczuk et al. 2011).

**Fig. 1:**
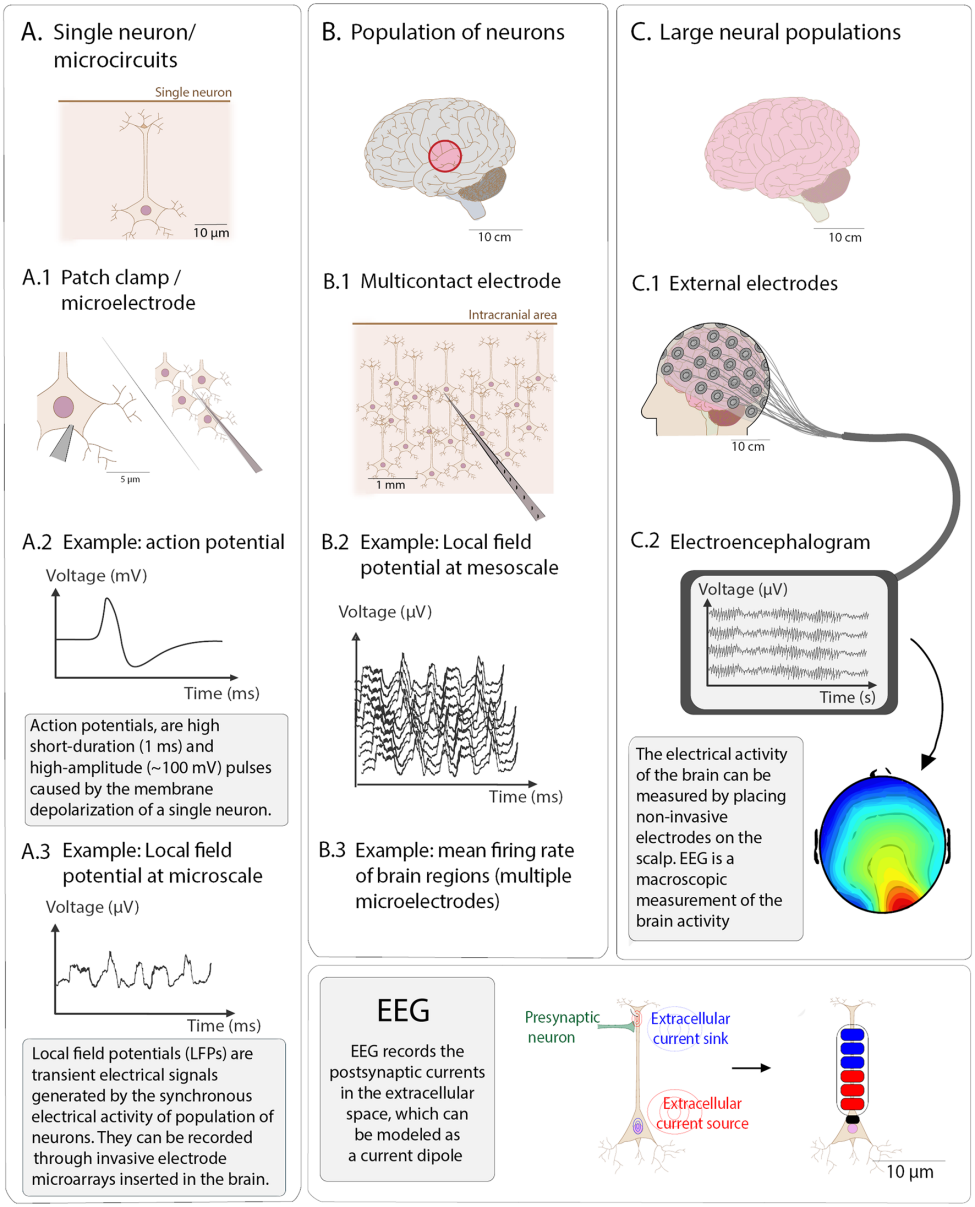
Electrophysiology of neural activity and EEG at different scales

The neurotransmitter release generated by action potentials, whether excitatory or inhibitory, results in local currents at the apical dendrites that in turn lead to current sources and sinks in the extracellular space around the dendritic arbor (i.e. postsynaptic potentials, see Fig. 1, bottom right block).

EEG shares sources with the local field potential (LFP), a low-pass filtered signal of extracellular measurements which represents the summed synaptic activity of local populations of neurons. In the neocortex, pyramidal neurons are generally organized perpendicularly to the cortical surface, with apical dendrites toward the pial surface and axons pointing inferiorly towards the grey-white matter border. This alignment leads to the electrical fields of many neurons being summed up to generate a signal that is measurable at the scalp (Tivadar and Murray 2019).

Importantly, individual neurons of these populations need to be (nearly) synchronously active to be detectable by EEG. When such large-scale synchronization occurs, it manifests in the EEG as *oscillations*, i.e. sustained sinusoidal activity with a characteristic frequency. The spectral properties of these oscillations—evaluated using the power spectrum—depend on cell types and their connectivity, but they also reflect the “brain state” (neurotransmitters, stimuli, disorders, etc.; see also section “Applications of computational models of EEG”) (Nunez et al. 2006).

As mentioned above, the electrical activity of the brain is recorded by means of electrodes, made of conductive materials, placed at the scalp. The propagation of electrical fields takes place due to the conductive properties of brain and head tissues, a phenomenon known as volume conduction (Kajikawa and Schroeder 2011). The electrodes are connected to an amplifier which boosts the signal. Due to the biophysical nature of what is measured, i.e. a voltage—the difference of potential able to move charges from one site to the other—EEG records the differential measurements between an electrode at a specific position on the scalp and a reference site. Common analyses in EEG are the study of local phenomena such as peaks at specific latencies or scalp sites (event-related potentials, ERPs); or the study of topography, i.e. the shape of the electric field at the scalp, which represents a global brain signature (Murray et al. 2008). EEG is known for its high temporal resolution. The biggest pitfalls of the technique are, on the other hand, the low spatial resolution and signal to noise ratio. A clear and exhaustive walk through these topics as well as an overview of strengths and pitfalls of using EEG is contained in Tivadar and Murray (2019) and for non-experts of the field in Biasiucci et al. (2019).

Despite being a measurement of the scalp activity, EEG can reveal the underlying neurophysiological mechanisms of the brain, and that is what classifies it as brain imaging tool. The estimation of the loci of active sources for the recorded brain activity at the scalp is called source reconstruction (Michel et al. 2004). However, the loci can belong to areas not necessarily below the considered electrode, a pitfall caused by volume conduction. Source reconstruction is a mathematically ill-posed inverse problem, as the solution is not unique. However, the addition of biophysical constraints to the inverse problem allows to retrieve a solution, which has been validated by means of intracranial recordings (Michel and Murray 2012). Having obtained the source activity, one can estimate the functional connectivity between the sources, i.e. the statistical dependencies between brain areas, assumed to indicate their interactions (see also Table 1). This can then be complemented with neuroanatomical/structural connectivity (Table 1), which estimates white matter connections between brain areas.

Computational models stand at the interface between the physiology of neurons at different scales (single neuron, population, macro-scale) and perceptual behavior. EEG would greatly benefit from the integration of in-silico simulations, as computational models could complement both the neurophysiological and behavioral interpretations of EEG recordings. In the following sections, we will discuss different types of computational models, i.e. the different scales at which the neural activity is simulated, how such models can be integrated in the analysis of EEG signals, and how such models have been used in new clinical applications.

## Different types of computational models for EEG

**Fig. 2:**
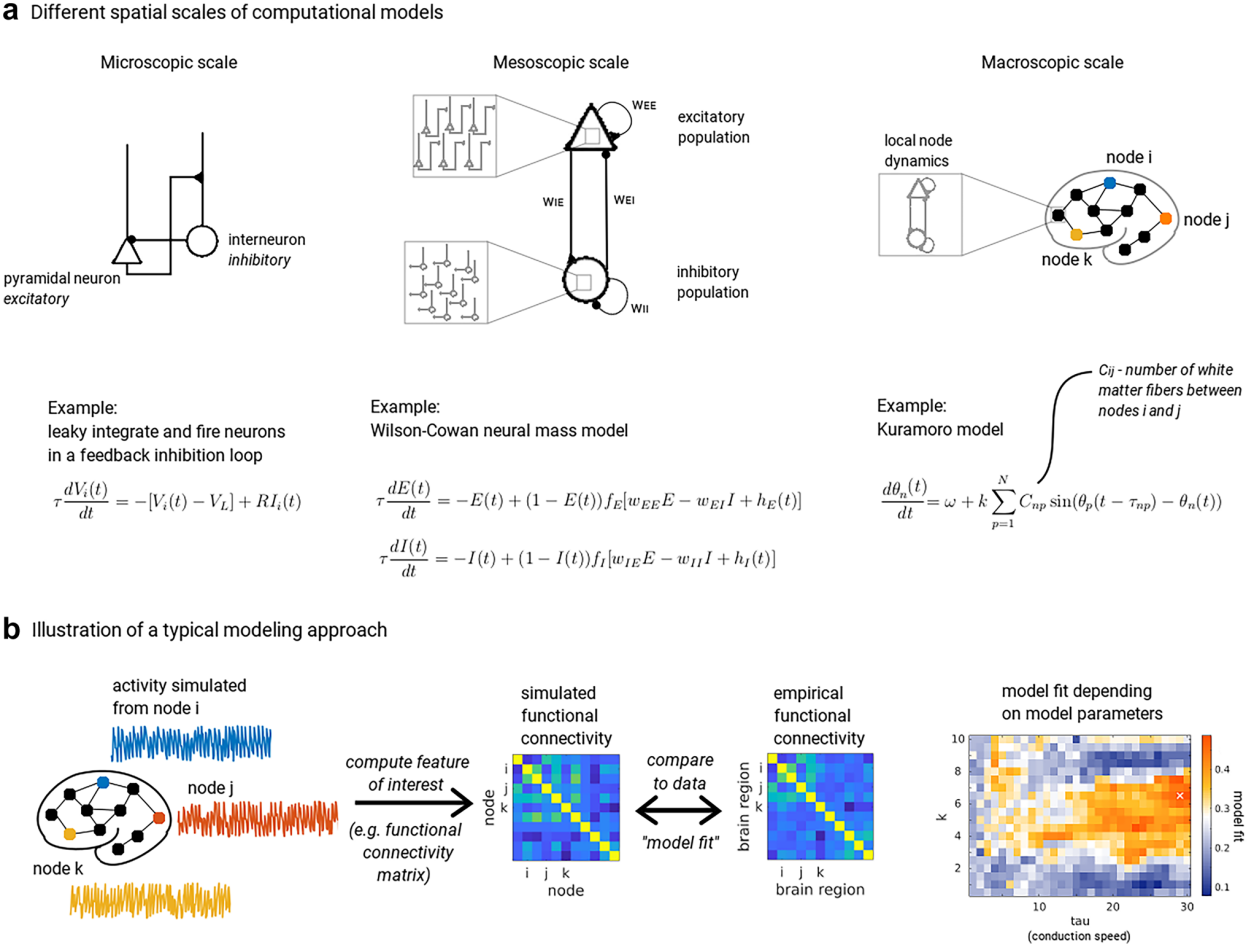
**a** Illustration of computational models at the three scales treated here. *Microscopic scale:* Simple example of two ($i=1,2$) leaky integrate-and-fire (LIF) neurons coupled together, a pyramidal neuron making an excitatory synapse to the interneuron, which in turn makes an inhibitory synapse to the pyramidal cell. This minimal circuit implements feedback inhibition, as the pyramidal cell, when activated, will excite the interneuron, which in turn will inhibit it. In the equation, $V_i$ is the membrane potential of each of the two cells $i = 1,2$; $V_L$ is the leak, or resting potential of the cells; *R* is a constant corresponding to the membrane resistance; $I_i$ is the synaptic input that each cell receives from the other, and possibly background input; $\tau$ is the time constant determining how quickly $V_i$ decays. The model is simulated by setting a firing threshold, at which, when reached, a spike is recorded and $V_i$ is reset to $V_L$. *Mesoscopic scale:* The Wilson–Cowan-model, in which an excitatory (*E*) and an inhibitory (*I*) population are coupled together. The mean field equations describe the mean activity of a large number of neurons. $f_E$ and $f_I$ are sigmoid transfer functions whose values indicate how many neurons in the population reach firing threshold, and $h_E$/$h_I$ are external inputs like background noise. $w_{EE}$ and $w_{II}$ are constants correponding to the strength of self-excitation/inhibition, and $w_{EI}$ and $w_{IE}$ the strength of synaptic coupling between populations. *Macroscopic scale*: In order to simulate long-range interactions between cortical and even subcortical areas, brain network models couple together many mesoscopic (“local”) models using the connection weights defined in the empirical structural connectivity matrix *C*. The example equation defines the Kuramoto model, in which the phase $\theta _n$ of each node *n* is used as a summary of its oscillatory activity around its natural frequency $\omega$. Each node’s phase depends on the phases of connected nodes *p* taking into account the time delay $\tau _{np}$, defined by the distances between nodes *n* and *p*. *k* is a global scaling parameter controlling the strength of internode connections. **b** Illustration of a typical modeling approach at the macroscopic scale. Activity is simulated for each node using the defined macroscopic model, e.g. the Kuramoto model from panel **a**, right. The feature of interest is then computed from this activity. Shown here is the functional connectivity, e.g., phase locking values between nodes (Table 1). This can then be compared to the empirical functional connectivity matrix computed in exactly the same way from experimental data, e.g. by correlating the entries of the matrix. The model fit can be determined depending on parameters of the model, e.g. the scaling parameter *k* or the unit speed, here indicated with “tau”

A straightforward classification of computational models for EEG can be done based on the different scales of neurophysiological activity they integrate. For instance, we can distinguish three types of models (Fig. 2a):

1. Microscopic models on the level of single cells and micro-circuits;
2. Mesoscopic models on the level of neural masses and neural fields;
3. Macroscopic models taking into account the connectome/white matter.

The integration of computational models has greatly advanced the field of applications of EEG, both for research and clinical purposes.

### Computational models for EEG on the level of single cells and microcircuits

The purpose of this level of modeling is to address the origin of the EEG signal by investigating the relationship between its features and electrophysiological mechanisms (Fig. 1, column A) with the tools of computational neuroscience. As detailed above, the EEG signal recorded from the scalp is the result of the spatial integration of the potential fluctuations in the extracellular medium. The EEG signal is mainly caused by the same mechanisms that generate the local field potential (LFP), i.e. it is driven by synaptic activity (Logothetis 2003; Buzsáki et al. 2012) and volume conduction (Kajikawa and Schroeder 2011). From the experimental standpoint, local network activity is usually measured as LFP (mainly in vivo—and rarely in vitro—animal data). By virtue of superposition, fluctuations in the LFP, and EEG more generally, are signatures of correlated neural activity (Pesaran et al. 2018). Cellular and microcircuit modeling are thus aimed at understanding the neurophysiological underpinnings of these correlations and the role played by cell types, connectivity and other properties in shaping the collective activity of neurons.

A primary goal of EEG modeling at the microscopic scale is on the one hand to predict the EEG signal generated by the summation of local dynamics on the microscopic scale and, on the other hand, to reconstruct the microscopic neural activity underlying the observed EEG. The first goal is far from being achieved, and the second is ill-posed due to the number of possible circuit and cellular combinations at the source level leading to similar EEGs. Implicit to these goals is to understand how features of neural circuits, such as the architecture, synapses and cell types, contribute to the generation of electromagnetic fields and their properties in a bottom-up fashion. Despite key insights, many shortcomings limit the interpretability of microcircuit models and the establishment of a one-to-one correspondence with EEG data. For instance, the contribution of spiking activity and correlated cellular fluctuations to LFPs and EEG power spectra remains unclear. Most microcircuit models characterize the net local network activity—used as a proxy for EEG—using the average firing rate or via the mean somatic membrane potential taken amongst populations of cells (of various types). Other studies have used a heuristic approach and approximated the EEG signal as a linear combination of somatic membrane potentials with random coefficients to account for both conduction effects and observational noise (Herrmann et al. 2016; Lefebvre et al. 2017). As such, microcircuit model predictions and experimental data cannot always be compared directly.

Cellular multicompartmental models, which oftentimes take cellular morphology and spatial configuration into consideration, are based on the celebrated Hodgkin-Huxley equations, which describe the temporal evolution of ionic flux across neuronal membranes (see Catterall et al. (2012), for a recent review). Such conductance-based models, which possess explicit and spatially distributed representations for cellular potentials, facilitate the prediction and/or comparison with LFP recordings. In contrast, single compartment models are difficult to interpret: while more abstract single compartment models such as Poisson neurons or integrate-and-fire models (Fig. 2a, left) are often used for their relative tractability and computational efficiency to construct more elaborate microcircuit models, they generally lack the neurophysiological richness to estimate EEG traces. Despite this, several computational advancements in recent years investigated how networks of integrate-and-fire neurons generate LFPs, clarifying the microscopic dynamics reflected in the EEG signal (Mazzoni et al. 2008, 2010, 2011, 2015; Deco et al. 2008; Buehlmann and Deco 2008; Barbieri et al. 2014). Such approaches have been used to understand the formation of correlated activity patterns in the hippocampus (e.g. oscillations), and their associated spectral fingerprints in the LFP (Chatzikalymniou and Skinner 2018). Furthermore, a broad range of works modeled the origin of the local field potential and how it diffuses via volume conduction to generate the EEG signal (Hindriks et al. 2017; Lindén et al. 2011; Mäki-Marttunen et al. 2019; Skaar et al. 2019; Einevoll et al. 2013; Teleńczuk et al. 2017; Bédard and Destexhe 2009).

The key missing element for understanding the link between spiking network activity, LFP, and EEG signal, is the functional and spatial architecture of the networks. In particular, there are two open challenges. The first is to understand how the network connectivity affects the model dynamics that generate the LFP, and the second is to clarify how the spatial arrangement and morphology of neurons affect LFP diffusion (Mazzoni et al. 2015).

From this perspective, models of pyramidal cell dynamics and circuits should guide the interpretation of the EEG signal. For example, Destexhe and colleagues recently addressed the long-debated issue of the relative contribution of inhibitory and excitatory signals to the extracellular signal (Teleńczuk et al. 2019), suggesting that the main source of the EEG signal may stem from inhibitory—rather than excitatory—inputs to pyramidal cells. A recent spiking network model (Saponati et al. 2019) incorporates the modular architecture of the thalamus, in which subnetworks connect to different parts of the cortex (Barardi et al. 2016). This model was used to show how the propagation of activity from the thalamus shapes gamma oscillations in the cortex.

Computational models at the level of single cells and microcircuits have also been instrumental in elucidating the mechanisms underlying multiple EEG phenomena. For instance, such models were used to better understand EEG rhythm changes observed before, during and after anesthesia, using spiking network models (McCarthy et al. 2008; Ching et al. 2012) and/or cortical micro-circuit models (Hutt et al. 2018). Some of these models have been extended to account for the effect of thalamocortical dynamics on EEG oscillations (Ching et al. 2010; Hutt et al. 2018), highlighting the key role played by the thalamus on shaping EEG dynamics. In addition, microcircuit models have been used to understand the EEG response of cortical networks to non-invasive brain stimulation (e.g. TACS, TMS), especially in regard to the interaction between endogenous EEG oscillatory activity and stimulation patterns (Herrmann et al. 2016), in which thalamic interactions were found to play an important role (Lefebvre et al. 2017).

### Computational models for EEG on the level of neural masses and neural fields

In this section we discuss models of population dynamics and how they could determine specific features of the electrical activity recorded by EEG (Fig. 1, column B). Mean field models describe the average activity of a large population of neurons by modeling how the population—as a whole—transforms its input currents into an average output firing rate (Fig. 2a, middle; for details on how networks of spiking neurons are reduced to mean field formulations, see Wong and Wang 2006; Deco et al. 2013b; Coombes and Byrne 2019; Byrne et al. 2020). If we consider a population to be a small portion of the cortex containing pyramidal cells, the average activity modelled by the mean field can be understood as the LFP. Two types of models can be distinguished: neural mass models, where variables are a function of time only, and neural field models, where variables are functions of time and space. In this sense, neural field models can be seen as an extension of neural mass models, by taking into account the continuous shape of cortical tissue and the spatial distribution of neurons. These models allow for the description of local lateral inhibition as well as local axonal delays (Hutt et al. 2003; Atay and Hutt 2006). An important application of neural field models is found in phenomenological models of visual hallucinations (Ermentrout and Cowan 1979; Bressloff et al. 2001), and they have been used to model sleep and anaesthesia (Steyn-Ross et al. 1999; Bojak and Liley 2005; Hindriks and van Putten 2012; Hashemi et al. 2015). Future applications may also involve both neural mass and neural field models to describe different cortical structures, similarly to the multiscale approach proposed in Cattani et al. (2016).

The most popular model on this mesoscopic scale was first described by Wilson and Cowan (Wilson and Cowan 1973; Cowan et al. 2016) (Fig. 2a, middle), and all mean field models can be seen as deriving from this form. It consists of an inhibitory and an excitatory population, where usually, for the purpose of EEG, it is assumed that the excitatory population models pyramidal neurons while the inhibitory population takes the role of interneurons. A variant of this model was described in Jansen and Rit (1995) and goes back to the “lumped parameter” model by Lopes Da Silva et al. (1974). It uses three distinct populations, i.e. a population of excitatory interneurons in addition to the two populations already mentioned. The reason this model has been popular in EEG modeling is that it accounts for the observation that inhibitory and excitatory synapses tend to deliver inputs to different parts of the pyramidal cell body (Sotero et al. 2007). In addition, thalamocortical loops are thought to greatly contribute to the generation of oscillations observed in the cortex (Steriade et al. 1993), and an important class of neural field models deals with these loops and their dependency on external stimuli (Robinson et al. 2001b, 2002).

The dynamical behavior of models can be manipulated to simulate different phenomena by varying their parameters. For example, the coupling parameters that determine the strength and speed of feedback-inhibition and feedforward-excitation can be varied (parameters $w_{IE}$ and $w_{EI}$ in Fig. 2a, middle), both within and between populations. Also it is possible to modify time constants (which govern the decay of activity in the local populations) or the strength of background noise. Changing these parameters in silico can be interpreted biologically. For example, in Bojak and Liley (2005), the authors describe how a modified neural field model reproduces EEG spectra recorded during anaesthesia. The strength of inputs from the thalamus to the cortical neural populations was varied within a biologically plausible range.

By coupling together more than one model/set of populations, one can start investigating the effect that delays have on neural activity (Jirsa and Haken 1996). In fact, Jansen and Rit (1995) coupled together two neural mass models in order to simulate the effect of interactions between cortical columns on their activity.

Neural mass and neural field models are able to reproduce a range of dynamical behaviors that are observed in EEG, like oscillations in typical EEG frequency bands (David and Friston 2003), phase-amplitude-coupling (Onslow et al. 2014; Sotero 2016), evoked responses (Jansen et al. 1993; Jansen and Rit 1995; David et al. 2005), and power spectra (Lopes Da Silva et al. 1974; Robinson et al. 2001b; David and Friston 2003; Bojak and Liley 2005; Moran et al. 2007). Power spectra are of particular interest in EEG because on the one hand, they can be precisely measured due to the high temporal resolution, and on the other, they can be thought of as a low-dimensional representation of steady-state dynamics. Consequently, much of EEG research focuses on studying shifts in the power spectrum due to task conditions, different cognitive states, or disorders. Of particular interest are linearized versions of these models, which make it possible to estimate the EEG spectrum in an analytical manner (Lopes Da Silva et al. 1974; Robinson et al. 2001b; Liley et al. 2002; Bojak and Liley 2005; Moran et al. 2007; Van Albada et al. 2010). Such solutions are not only more easily interpretable in terms of the impact of varying different parameters and more computationally efficient. They also allow the researcher to quantify the contribution of nonlinearities to the observed power spectra, thus tackling the question of which level of complexity is necessary in computational modeling of EEG.

Often, activity simulated by mean field models is assumed to be related to local field potentials (Liley et al. 2002). However, models are usually set up such that the local field potential derives directly from the mean firing rate. In this way, an important aspect that underlies the EEG signal is neglected, namely, the synchrony (coherence) of the firing within a neural population (as opposed to synchrony between populations, which can be studied using e.g. instantaneous phase differences (Breakspear et al. 2004)). Phenomena such as event-related synchronization and -desynchronization result from a change in this synchrony rather than from a change in firing rate. Recent models (Byrne et al. 2017, 2020) propose therefore a link between the firing rate and the Kuramoto order parameter, which is a measure of how dispersed firing is within a population.

### Macroscopic computational models for EEG taking into account the connectome

In this section, we review existing literature on macroscopic computational models that take into account the connectome and discuss their potential to reveal the generative mechanisms of the macroscopic brain activity patterns detected with EEG and MEG (Fig. 1, column C). We will use the term “brain network models” (BNM) in order to clearly distinguish this framework from other approaches to whole-brain modeling (Breakspear 2017), e.g. using neural field models (Jirsa and Haken 1996; Robinson et al. 1997; Coombes et al. 2007) or expansions of the thalamocortical models discussed above (Robinson et al. 2001b; Freyer et al. 2011). We will also leave aside the large body of literature on dynamic causal modeling (DCM) (Kiebel et al. 2008; Pinotsis et al. 2012), as this deserves a more detailed review than the scope of this paper can provide.

*Brain network models.* In recent years, the interest in the human connectome has experienced a boom, creating the prolific and successful field of “connectomics”. In the framework of connectomics, the brain is conceptualized as a network made up of nodes and edges. Each node represents a brain region, and nodes are coupled together according to a weighted matrix representing the wiring structure of the brain (Fig. 2a, right). This so-called structural connectivity matrix (SC) is derived from white matter fiber bundles which connect distant brain regions (Behrens et al. 2003; Zhang et al. 2010; Hagmann et al. 2008; Sepulcre et al. 2010; Wedeen et al. 2012) and are measured using diffusion weighted magnetic resonance imaging (dMRI) (Table 1). The set of all fiber bundles is called the connectome (Sporns 2011). In practice, brain regions are defined according to an existing brain atlas (for example Desikan et al. (2006); Hagmann et al. (2008); Glasser et al. (2016)), and the weights in the SC matrix are taken as the fiber count (number of streamlines found by a fiber tracking algorithm), fiber density (number of streamlines divided by region size), or, less commonly, some other diffusion imaging-derived quantity, e.g. fractional anisotropy (Wedeen et al. 2008; Iturria-Medina et al. 2008; Essayed et al. 2017). By coupling brain regions together according to the weights in the SC, the activity generated in each region depends also on the activity propagated from other regions along the connections given by the SC. Note that in the previous section, we already mentioned the possibility of coupling local populations. However, in those cases, the coupling is usually determined by a kernel which defines a dependency of the coupling on the geodesic distance between populations.

BNMs are used to study the role of structural connectivity in shaping brain activity patterns. Because this is a complex problem that involves the entire brain, it is important to find a balance between realism and reduction, so that useful predictions can be made. In practice, a common simplification is to assume that all brain regions are largely identical in their dynamical properties (Passingham et al. 2002). This *reductionist* approach keeps the number of parameters at a manageable level and still allows to investigate how collective phenomena emerge from the *realistic* connectivity between nodes. In other words, BNMs do not necessarily aim at maximizing the fit to the empirically recorded brain signals. Rather, the goal is to reproduce specific temporal, spatial or spectral features of the empirical data emerging at the macroscopic scale whose underlying mechanisms remain unclear (Fig. 2b).

*Choice of local model.* In mathematical terms, brain activity is simulated according to a system of coupled differential equations. The activity of each node is described by a mean-field model, such as the ones described in the section “Computational models for EEG on the level of single cells and microcircuits”, and coupling between the mean field models is parametrized by the empirical SC (Fig. 1a, right).

Importantly, the type of mean-field model used at the local level must be selected according to the hypothesis being tested. For example, BNMs have proved to be a powerful tool to elucidate the non-linear link between the brain’s structural wiring and the functional patterns of brain activity captured with resting-state functional magnetic resonance imaging (rsfMRI) (Deco et al. 2013a, 2014a; Honey et al. 2009; Deco et al. 2009; Cabral et al. 2011). However, oscillations in frequency ranges important for M/EEG (2–100 Hz) are often neglected in studies aiming at reproducing correlated fluctuations on the slow time scale of the fMRI signal. Thus, despite the insights gained by BNMs to understand rsfMRI signal dynamics, the same models do not necessarily serve to understand M/EEG signals and vice-versa (Cabral et al. 2017).

In Cabral et al. (2014), the local model employed includes a mechanism for the generation of collective oscillatory signals in order to address oscillatory components of M/EEG. To model brain-wide interactions between local nodes oscillating around a given natural frequency (in this case, 40 Hz, in the gamma frequency range), the Kuramoto model (Kuramoto 2003; Yeung and Strogatz 1999), was extended to incorporate realistic brain connectivity (SC) and time delays (determined by the lengths of the fibers in the SC (see also Finger et al. (2016); Fig. 2a, right). This model shows how, for a specific range of parameters, groups of nodes (communities) can temporarily synchronize at community-specific lower frequencies, obeying to universal rules that govern the behaviour of coupled oscillators with time delays. Thus, the model proposed a mechanism that explains how slow global rhythms in the alpha- and beta-range emerge from interactions of fast local (gamma) oscillations generated by neuronal networks.

In contrast, Deco et al. (2019b) used a mean field model (Wilson and Cowan 1973; Brunel and Wang 2001; Deco and Jirsa 2012; Deco et al. 2014b), which was tuned not to exhibit intrinsic oscillations. Because the brain could thus be considered as being in a noisy, low-activity state, the number of parameters was sufficiently reduced to investigate how activation patterns change over time on different time scales. Time scales including that of M/EEG (ten to several 100 ms) as well as that typical for fMRI (1–3 s) were considered, and the question was asked whether there is a time scale at which brain dynamics are particularly rich. The authors found that both the number of co-activation patterns as well as their dynamics were richest when a time scale of 200 ms was used. Thus, in this case, co-activation patterns were of interest instead of oscillations, and a model suitable for both M/EEG and fMRI was chosen.

*Emerging class of harmonics-based models.* Although both the described BNMs as well as DCM (dynamic causal modeling) have a long history of success in modeling brain activity patterns, they have high-dimensionality, and usually require local oscillators governed by region-specific or spatially-varying model parameters. While this imbues such models with rich features capable of recreating complex behavior, they are challenging for some clinical applications where a small set of global features might be desired to assess the effect of disease on network activity.

Nunez (1974) presented pioneering modeling work that focuses on the global aspects of brain dynamics, which was continuously developed over the last decades (Nunez 1974, 1989; Nunez and Srinivasan 2006; Nunez et al. 2019). The idea at the basis of these models is that global brain dynamics can be understood as standing and traveling waves constrained by the brain geometry, an idea that remains immensely influential.

In order to take advantage of the low-dimensional properties of such models, some laboratories have recently focused on low-dimensional processes involving diffusion or random walks (Table 1) on the structural graph (Table 1) instead of mean-field models, providing a simpler means of simulating functional connectivity (FC). These simpler models were able to match or exceed the predictive power of complex neural mass models or DCMs in predicting empirical FC (Abdelnour et al. 2014). Higher-order walks on graphs have also been quite successful; typically these methods involve a series expansion of the graph adjacency or Laplacian matrices (Meier et al. 2016; Becker et al. 2018) (Table 1). Not surprisingly, the diffusion and series expansion methods are closely related, and most of these approaches may be interpreted as special cases of each other, as demonstrated elegantly in recent studies (Robinson et al. 2016; Deslauriers-Gauthier et al. 2020; Tewarie et al. 2020).

Whether using graph diffusion or series expansion, these models of spread naturally employ the so-called eigenmodes, or harmonics, of graph adjacency or Laplacian matrix. Hence these methods were generalized to yield spectral graph models whereby e.g. Laplacian harmonics were sufficient to reproduce empirical FC, using only a few eigenmodes (Galán 2008; Atasoy et al. 2016; Abdelnour et al. 2018). The Laplacian matrix in particular has a long history in graph modeling, and its eigenmodes are the orthonormal basis of the network and can thus represent arbitrary patterns on the network (Stewart 1999). Such spectral graph models are computationally attractive due to low-dimensionality and more interpretable analytical solutions. The SC’s Laplacian eigenmodes may be thought of as the substrate on which functional patterns of the brain are established via a process of network transmission (Abdelnour et al. 2018; Atasoy et al. 2016; Robinson et al. 2016; Preti and Van De Ville 2019; Glomb et al. 2020). These models were strikingly successful in replicating canonical functional networks, which are stable large scale circuits made up of functionally distinct brain regions distributed across the cortex that were extracted by clustering a large fMRI dataset (Yeo et al. 2011).

While spectral graph models have demonstrated ability to capture essential steady-state, stationary characteristics of real brain activity, they are limited to modeling passive spread without oscillatory behavior. Hence they may not suitably accommodate a larger repertoire of dynamically-varying microstates or rich power spectra at higher frequencies typically observed on EEG or MEG. Capturing the rich repertoire of brain dynamics would require a full accounting of axonal propagation delays as well as local neural population dynamics within graph models, as previously advocated (Cabral et al. 2011). In O’Connor et al. (2002); O’Connor and Robinson (2004), the authors used neural field models (Robinson et al. 2001a) to derive relationships between wave patterns on the cortical sheet and EEG power spectra. This was later extended to spherical geometries (Robinson et al. 2016; Mukta et al. 2017). Roberts et al. (2019) explored traveling waves on the network derived from the SC, and Tewarie et al. (2019) successfully modeled band-specific MEG resting-state networks with a combination of delayed neural mass models and eigenmodes of the structural network (Tewarie et al. 2019), suggesting delayed interactions in a brain’s network give rise to functional patterns constrained by structural eigenmodes.

Recently another effort was undertaken to characterize wide-band brain activity using graph harmonics in closed form (i.e. requiring no time-domain simulations), a rarity in the field of computational neuroscience (Raj et al. 2020). This “spectral graph model” of brain activity produced realistic power spectra that could successfully predict both the spatial as well as temporal properties of MEG empirical recordings (Raj et al. 2020). Intriguingly, the model has very few (six) parameters, all of which are global and not dependent on local oscillations. This method therefore exemplifies the power of graph methods in reproducing more complex and rich repertoire of brain activity, while keeping to a parsimonious approach that does not require the kinds of high-dimensional and non-linear oscillatory models that have traditionally held sway.

## Applications of computational models of EEG

Network oscillations captured through EEG are thought to reflect relevant processes for brain function, namely for cognition, memory, perception, and consciousness (Ward 2003). Indeed, oscillatory activity in EEG signals is found to change with a wide range of tasks and to exhibit characteristic features across states of consciousness. Moreover, alterations in oscillatory activity can be a sign of a brain disorder, with EEG commonly used in research and clinical fields to help diagnosis and treatment (Tatum 2014). It is known that coherence across sufficiently large brain regions is necessary for oscillations to be detectable with EEG. However, the mechanisms generating and orchestrating these oscillations at the mesoscopic and macroscopic levels remain mostly unclear. Following different mechanistic scenarios, physiologically and/or theoretically inspired computational models have been shown to reproduce characteristic features of EEG signals, offering a complementary tool to address healthy and disease brain mechanisms, test new clinical hypotheses, and explore new surgical strategies in silico. This section presents a number of computational works that used mostly large-scale network approaches to explain the changes in brain activity observed across the spectrum of consciousness as well as in neuropsychiatric disorders and epilepsy.

### Brain models of consciousness

A variety of models have been employed to elucidate the neurophysiological mechanisms underlying different physiological brain states, such as wakefulness and deep sleep (non-rapid eye movement, NREM) (Robinson et al. 2002; Hill and Tononi 2005; Roberts and Robinson 2012; Cona et al. 2014), and pharmacological conditions, such as anesthesia (Steyn-Ross et al. 1999; Sheeba et al. 2008; Ching et al. 2010; Hutt and Longtin 2010; Liley et al. 2010; Hindriks and van Putten 2012; Ching and Brown 2014; Hashemi et al. 2015). Contrasting the activity detected in quiet wakeful rest with the activity detected in deep sleep and/or anesthesia has been the subject of research of a number of computational models addressing signatures of consciousness captured both with EEG (Esser et al. 2009; Ching et al. 2010) and fMRI (Deco et al. 2018, 2019a). In fact, understanding the mechanisms underlying consciousness might have crucial implications for the study of disorders of consciousness (DoC). DoC refer to a class of clinical conditions that may follow a severe brain injury (hypoxic/ischemic or traumatic brain injury) and include coma, vegetative state or unresponsive wakefulness syndrome (VS/UWS), and minimally conscious state (MCS). Coma has been defined as a state of unresponsiveness characterized by the absence of arousal (patients lie with their eyes closed) and, hence, of awareness. VS/UWS denotes a condition of wakefulness with reflex movements but without behavioural signs of awareness, while patients in MCS show unequivocal signs of interaction with the environment.

The current gold standard for clinical assessment of consciousness relies on the Coma Recovery Scale Revised (Giacino et al. 2004), which scores the ability of patients to behaviourally respond to sensory stimuli or commands. However, behavioral-based clinical diagnoses can lead to misclassification of MCS as VS/UWS because some patients may regain consciousness without recovering their ability to understand, move and communicate (Childs et al. 1993; Andrews et al. 1996; Schnakers et al. 2009). A great effort has been devoted to develop advanced imaging and neurophysiological techniques for assessing covert consciousness and to improve diagnostic and prognostic accuracy (Edlow et al. 2017; Bodart et al. 2017; Stender et al. 2014; Bruno et al. 2011; Owen and Coleman 2008; Stender et al. 2016). A novel neurophysiological approach to unravel the capacity of the brain to sustain consciousness exploits Transcranial Magnetic Stimulation (TMS) in combination with EEG (Rosanova et al. 2018; Casarotto et al. 2016). Specifically, the EEG response evoked by TMS in *conscious* subjects exhibits complex patterns of activation resulting from preserved cortical interactions. In contrast, when *unconscious* patients are stimulated with TMS, the evoked-response shows a local pattern of activation, similar to the one observed in healthy controls during NREM sleep and anesthesia.

The perturbational complexity index (PCI) (Casali et al. 2013) is an electroencephalographic-derived measure that quantifies the dynamical complexity of TMS-evoked EEG potentials by means of the Lempel-Ziv compression algorithm, showing high values (low compressibility) for complex chains of activation typical of the awake state, and low values (high compressibility) for stereotypical patterns of activation typical of sleep and anesthesia. PCI has been validated on a benchmark population of 150 conscious and unconscious controls and tested on 81 severely brain-injured patients (Casarotto et al. 2016), showing an unprecedented high sensitivity (94.7%) in discriminating conscious from unconscious states.

Although PCI is commonly used to analyze real TMS-EEG data, it can also be applied to simulated data (Bensaid et al. 2019). This recently published modeling approach investigates the physiological mechanisms underlying the generation of complex or stereotypical TMS-evoked EEG responses. The proposed brain network model, named COALIA in Bensaid et al. (2019), describes local dynamics as neural masses (Wendling et al. 2002) that include populations of pyramidal neurons and three different types of interneurons. Each neural mass describes the local field activity of one of 66 cortical brain regions (Desikan et al. 2006). Neural masses are connected with each other through long-range white matter fibers as described above (section “Macroscopic computational models for EEG taking into account the connectome”). EEG signals are then simulated as neural mass activity. A systematic comparison of the complexity of simulated and real TMS-evoked EEG potentials through PCI suggested that the rhythmically patterned thalamocortical activity, typical of sleep, plays a key role in disrupting the complex patterns of activation evoked by TMS (Bensaid et al. 2019). Indeed, this rhythmical thalamocortical activity results in inhibition within the cortex that prevents information from propagating from one brain region to another, and thus disrupts functional integration, i.e. the ability of the brain to integrate information originating from different brain regions or groups of brain regions (Tononi 1998). Along with functional segregation, i.e., the specialization of a brain region to fulfill a certain function, functional integration is necessary to generate complex time-varying patterns of coordinated cortical activity that are typical of the awake brain, and thought to sustain consciousness and cognitive functions (Tononi et al. 1994; Casali et al. 2013; Lord et al. 2017; Demertzi et al. 2019).

### Neuropsychiatric disorders

Disruption of integration and segregation balance, which is fundamental for consciousness as mentioned in the section “Brain models of consciousness”, have been linked also to several neuropsychiatric disorders as a result of altered structural and functional connectivity (Bassett and Bullmore 2009; Fornito et al. 2015; Menon 2011; Deco et al. 2015). Among neuropsychiatric disorders, as reviewed in Lord et al. (2017), Alzheimer’s disease is characterized by a decrease in long-range functional connectivity, directly affecting integration between functional modules of the brain (Stam et al. 2007; Sanz-Arigita et al. 2010). Schizophrenia has been linked to a “subtle randomization” of global functional connectivity, such that the so-called “small-world” character of the network is disrupted (Alexander-Bloch et al. 2010; Lynall et al. 2010); a small-world network is characterized by short path lengths and strong modularity, network properties that are thought to promote information processing in the brain (Bassett and Bullmore 2006) (but see Hilgetag and Goulas (2016)). Loss of integration has also been observed in schizophrenia (Damaraju et al. 2014).

As explained in the section “Macroscopic omputational models for EEG taking into account the connectome”, whole-brain computational models provide insights into how anatomical connections shape and constrain functional connectivity (Honey et al. 2009; Deco et al. 2013a, 2014a). Using BNMs, Cabral and colleagues have shown that the alterations reported in schizophrenia (Lynall et al. 2010) can be explained by a decrease in connectivity between brain areas, occurring either at the local or global level and encompassing either axonal or synaptic mechanisms, hence reinforcing the idea of schizophrenia being the behavioural consequence of a multitude of causes disrupting connectivity between brain areas (Cabral et al. 2012a, b).

However, these models have focused on reproducing fMRI findings and are yet to be extended to address alterations in EEG spectral signatures in schizophrenia, namely increased EEG gamma-band power and decreased alpha power (Uhlhaas and Singer 2013), which, following previous modeling insights (Cabral et al. 2014), may arise from reduced coupling between local gamma-band oscillators. Furthermore, BNMs can be employed to test how clinical interventions may help to re-establish healthy network properties such as the balance between integration and segregation or small-worldness (Deco et al. 2018, 2019a).

### Epilepsy

Models have been employed to study pathological alterations of network oscillatory activity related to many diseases, including epilepsy (Wendling 2005; Lytton 2008; Stefanescu et al. 2012; Holt and Netoff 2013). Epilepsy is a complex disease which impacts 1% of the world population and is drug resistant in approximately 30% of cases. Due to its intrinsic complexity, epilepsy research has strongly benefited, and will do so even more in the future, from an in silico environment where hypotheses about brain mechanisms of epileptic seizures can be tested in order to guide strategies for surgical, pharmacological and electrical stimulation techniques.

Focal epilepsy is a prototypical example of a disease that involves both local tissue and network properties. Focal epilepsy occurs when seizures originate in one or multiple sites, so-called epileptogenic zones (EZ), before recruiting close and distal non-epileptogenic areas pertaining to the pathological network. Patients with a history of drug-resistant focal epilepsy are candidates for surgery which targets epileptogenic areas and/or critical nodes presumably involved in the epileptogenic network. Successful outcomes of these procedures critically rely on the ability of clinicians to precisely identify the EZ.

A promising modeling approach aims at studying focal epilepsy through a single-subject virtual brain (Soltesz and Staley 2011; Terry et al. 2012; Hutchings et al. 2015; Proix et al. 2017; Bansal et al. 2018), bringing together the description of how seizures start and end (seizure onset and offset, respectively) at a local level (through neural mass models) (Robinson et al. 2002; Wendling et al. 2002; Lopes da Silva et al. 2003; Breakspear et al. 2006; Jirsa et al. 2014) with individual brain connectivity derived from dMRI data. In this personalized approach, a patient’s brain is virtually reconstructed, such that systematic testing of many surgical scenarios is possible. The individual virtual brain approach provides clinicians with additional information, helping them to identify locations which are responsible for starting or propagating the seizure and whose removal would therefore lead to the patient being seizure-free while avoiding functional side effects of removing brain regions and connections (Proix et al. 2017; Olmi et al. 2019). Finally, in silico approaches involving neurostimulation paradigms provide useful insights about how to prevent seizure onset or to interrupt the propagation of partial seizure to large brain areas (Schiff 2012; Stamoulis 2013; Rich et al. 2020).

**Table 1:** Some terminology used in this paper

Functional connectivity	(FC) Statistical dependencies between time series recorded from different brain regions or simulated at different nodes. Such dependencies are taken to indicate a functional relatedness of the brain regions/nodes. Many measures are available, for example correlation between amplitude envelopes, phase locking value, imaginary coherence, etc. See for example Colclough et al. (2016) for an overview. Note that FC does not establish a causal relationship (Friston 2011)
Structural connectivity	(SC) Also known as neuroanatomical, anatomical, or white matter connectivity. Diffusion-weighted MRI (dMRI) is able to measure the diffusion of water through brain tissue (Basser et al. 1994). As water diffuses preferably *along* axons rather than across their walls, the orientation of large fiber bundles can be inferred from dMRI via algorithms known as fiber tracking (Jones 2010). Note that SC does not take into account local anatomical connections made within the gray matter, and that fiber counts or densities do not allow making conclusions about the weight of that connection (Jeurissen et al. 2019). Furthermore, fiber tracking algorithms are unable to resolve ambiguities introduces by crossing fibers, and it is difficult to track long fibers
Graph	A brain network model, which consists of nodes and edges (Fig. 2a, right), can be formalized as a graph (Bassett and Sporns 2017; Sporns 2018). This can be visualized using so-called adjacency matrices, which contain a weight for the edge between each pair of nodes (Fig. 2b). In this sense, both FC and SC matrices are adjacency matrices. This formalization opens up the analysis of brain networks to the tools of graph analysis. These tools allow for example the characterization of the graph/network using many different quantitative measures (Rubinov and Sporns 2010), partitioning the graph/network into subnetworks or modules (Bassett and Sporns 2017; Donetti and Munoz 2004), or classifying nodes depending on their role in the network (Hagmann et al. 2008)
Random walk	A random walk is a random process taking place on the graph in which a “walker” is initiated at a node and proceeds to another node following existing edges. Edges are selected by the walker with a probability proportional to their weight. Such a simulation can be used to approximate the dynamics of spreading activation, and enables the researcher to approximate for example the probability that activity will spread from node *i* to node *j* given the edges that exist between them, or the time that it will take for activity to spread from node *i* to node *j*
Laplacian	The Laplace operator is ubiquitous in many physical systems and is used to describe standing waves, diffusion, heat dispersion, and many other phenomena. For a network, the Laplacian is obtained from the functional or structural connectivity matrix (see above). An intuitive interpretation is that it describes the “flow” of activity along the edges
Eigenmodes	Many physical systems that consist of interacting elements can vibrate at certain frequencies, for example the string of a violin or the vibrating sheets of Chladni (1802). Each system has its own set of frequencies at which it can vibrate, determined for example by its shape. Mathematically, these eigenmodes are obtained via eigendecomposition of the Laplacian (see above)

## Discussion

In this paper we introduced different computational model types and their application to EEG, using a simple classification by spatial scale. Clearly not all models in the literature would necessarily belong to one category, but we believe this taxonomy can provide an entry point for non-experts. The main motivation behind this review was to identify obstacles that stand in the way of applying EEG modeling in both a research and clinical context, and to point out future directions that could remove these obstacles.

We have pointed out several recent efforts that have begun to more closely align models and experimental findings. Such integration of theory and experiment guarantees the use of biologically relevant measures within computational models of EEG, a crucial element if one wishes to use EEG models together with acquired data. For example, recent microcircuit models address the gap between theory and experiment by linking average firing rate—a measure of population activity preferred by the modeling community—and local field potential (LFP)—a measure that is generally thought to be a good proxy of the EEG signal (Saponati et al. 2019); recent mean field models explicitly include the contribution of neural synchronization to the LFP (Byrne et al. 2020), thereby integrating experimental knowledge about how the EEG signal is generated (Lopes da Silva 2013); brain network models explore the contribution of empirically measured connectomes to macroscopic brain activity (Cabral et al. 2014); and applications of computational models already exist that use clinical measures to study e.g. coma (Bensaid et al. 2019), epilepsy (Olmi et al. 2019; Proix et al. 2017), and neuropsychiatric disorders (Spiegler et al. 2016; Kunze et al. 2016). Furthermore, some modeling approaches focus on providing a simple model for large-scale dynamics, making results more interpretable both from a theoretical and clinical standpoint (Abdelnour et al. 2018; Raj et al. 2020).

We have reviewed computational models on three spatial scales (Fig. 2a). Each scale models qualitatively different biological processes which can be measured using distinct recording techniques (Varela et al. 2001) (Fig. 1). While EEG records activity at the macro-scale, mechanisms at each scale have an impact on the EEG signal and should therefore inform its interpretation. Therefore, ideally, scales should be combined to provide a complete picture of neural mechanisms underlying EEG activity, something that started to be explored for example in the simulation platform The Virtual Brain (TVB) (Sanz Leon et al. 2013; Falcon et al. 2016) or in studies showing the theoretical relationship between spiking networks and mean field formulations (Wong and Wang 2006; Deco et al. 2013b; Coombes and Byrne 2019; Byrne et al. 2020). Using models in this hierarchical manner is the only way to disentangle different contributions to the EEG signal without using invasive techniques, i.e., to distinguish neural signals (Michel and Murray 2012; Seeber et al. 2019), volume conduction (Hindriks et al. 2017; Lindén et al. 2011; Mäki-Marttunen et al. 2019; Skaar et al. 2019; Einevoll et al. 2013; Teleńczuk et al. 2017; Bédard and Destexhe 2009), and noise. Furthermore, brain disorders can impact brain structure and function on any scale. Using models on multiple scales is necessary if one wishes to understand how pathological changes manifest in clinically measurable EEG signals. Such an understanding would also allow to use EEG to evaluate clinical interventions that affect the micro- or mesoscale (e.g., drugs).

Models can thus play an important role as a “bridge” that connects different fields. In translational applications, knowledge from basic research can be integrated into a model and the model can be designed in such a way that it is useful for a clinical application. An example for a successful “bridge” is the case of Brain Computer Interfaces. In order to realize multi-scale models, researchers working on animal recordings and researchers focusing on non-invasive recordings in humans have to come together with modeling experts that can incorporate findings from both fields in a model.

As an outlook, EEG modeling could play an important part in future endeavors towards precision medicine, or “personal health”. Individual brain models could be used to integrate different sources of data (EEG, fMRI, ECG, etc.) in a “virtual patient”. This could complement data-driven approaches like connectome fingerprinting [in which individuals are identified using their individual connectome (Finn et al. 2015; Pallarés et al. 2018; Abbas et al. 2020)]. The ultimate goal would be to use this virtual patient to tailor diagnosis and therapies around the needs of the patient (Wium-Andersen et al. 2017), reducing the economical burden and patient discomfort of clinical analyses and hospitalization.
